# Supplementary material for: An RNA-Based Vaccine Platform for Use against Mycobacterium tuberculosis
Source: Vaccines (Basel). 2023 Jan 5;11(1):130. doi: 10.3390/vaccines11010130 (PMC9862644; doi:10.3390/vaccines11010130)
Supplement: Supplementary file 1 [file vaccines-11-00130-s001.zip › vaccines-2022749-supplementary.pdf]

Supplemental Figures

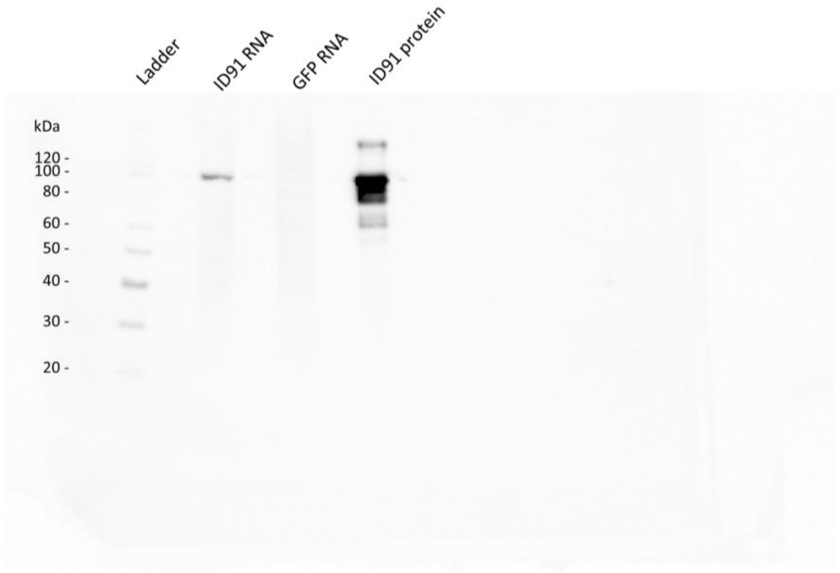

Supplemental Figure S1: Uncropped Western Blots using-ID91 polyclonal sera. Cropped blot image referenced in Figure 1.

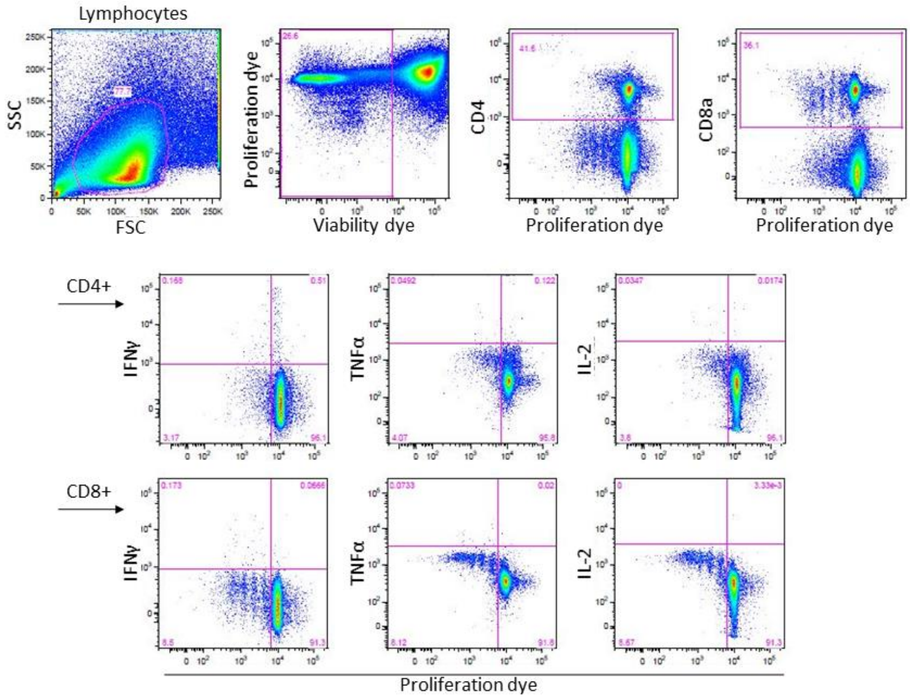

Supplemental Figure S2: Representative Gating for Epitope Mapping. C57BL/6 mice were immunized twice, three weeks apart with ID91 repRNA + NLC (RNA) or ID91+GLA-SE. Two weeks post boost splenocytes were stimulated with media, whole antigen, or 13 different peptide pools (PP) with overlapping 15mers of ID91. Proliferation and cytokine expression were measured by flow cytometry after stimulation. Data shown are representative of PP stimulation and subsequent gating of viable lymphocytes, proliferation and IFNγ, TNFα and IL-2 cytokine expression.

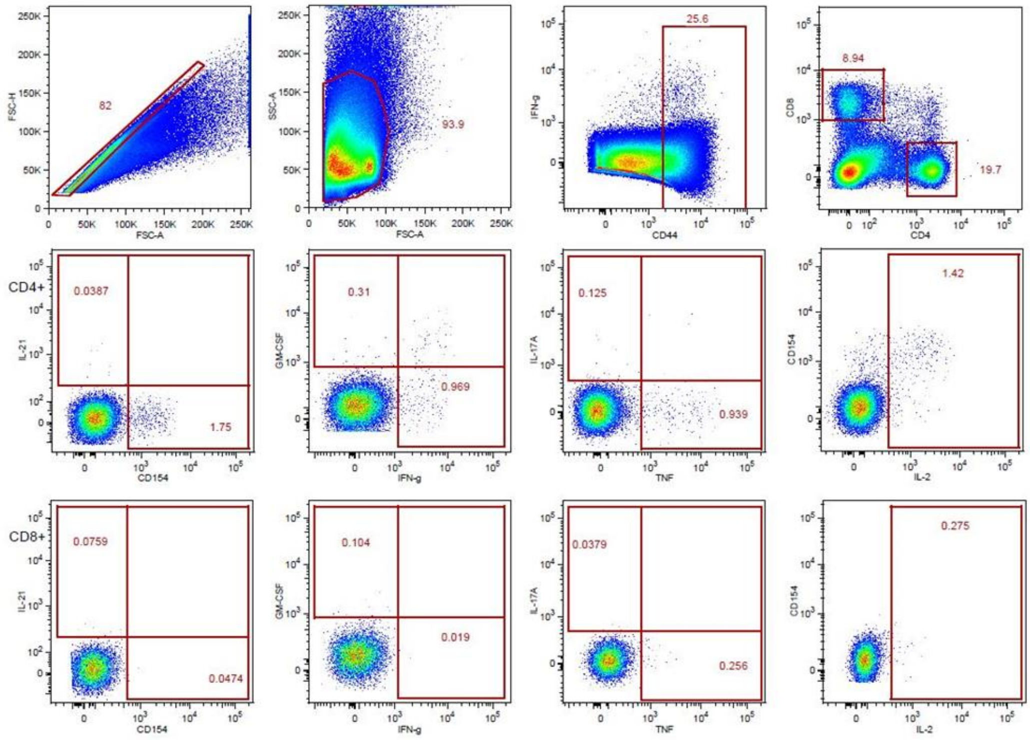

Supplemental Figure S3: Representative Gating for Pre-challenge Immunogenicity. Splenocytes from cohorts of saline, ID91+GLA-SE or ID91 repRNA + NLC immunized mice 6 weeks post-vaccination were stimulated with ID91 *ex vivo* and evaluated by intracellular cytokine staining flow cytometry including percentage of CD4+ CD44+ cytokine producing T cells as well as percentage of CD8+ CD44+ cytokine producing T cells after medium subtraction. Data representative ID91+GLA-SE immunized mouse stimulated with ID91 antigen *ex vivo* and subsequent gating of viable lymphocytes, and IFNγ, GMCSF, IL-17A, CD154, TNFα, IL-21 and IL-2 cytokine expression.

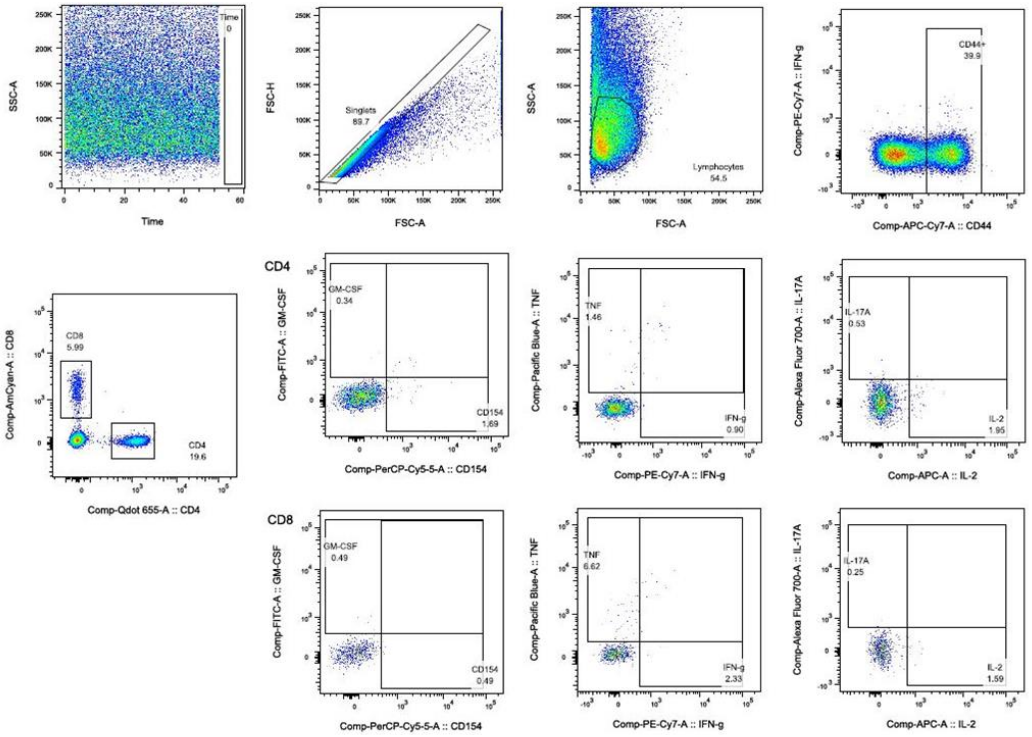

Supplemental Figure S4: Representative Gating for post-challenge Immunogenicity. Cells from lung homogenates from cohorts of saline, ID91+GLA-SE or ID91 repRNA + NLC immunized mice 3 weeks post-vaccination were stimulated with ID91 *ex vivo* and evaluated by intracellular cytokine staining flow cytometry including percentage of CD4+ CD44+ cytokine producing T cells as well as percentage of CD8+ CD44+ cytokine producing T cells after medium subtraction. Data representative of combination immunized mouse stimulated with ID91 PP *ex vivo* and subsequent gating of singlets, lymphocytes, and IFNγ, GMCSF, IL-17A, CD154, TNFα, and IL-2 cytokine expression.
